# Transcriptional Profiling of Porcine HCC Xenografts Provides Insights Into Tumor Cell Microenvironment Signaling

**DOI:** 10.3389/fgene.2021.657330

**Published:** 2021-04-29

**Authors:** Shovik S. Patel, Amitha Sandur, Mohammed El-Kebir, Ron C. Gaba, Lawrence B. Schook, Kyle M. Schachtschneider

**Affiliations:** ^1^Department of Radiology, University of Illinois at Chicago, Chicago, IL, United States; ^2^Department of Computer Science, University of Illinois at Urbana-Champaign, Urbana, IL, United States; ^3^Department of Animal Sciences, University of Illinois at Urbana-Champaign, Urbana, IL, United States; ^4^National Center for Supercomputing Applications, University of Illinois at Urbana-Champaign, Urbana, IL, United States; ^5^Department of Biochemistry and Molecular Genetics, University of Illinois at Chicago, Chicago, IL, United States

**Keywords:** hepatocellular carcinoma, hepatic fibrosis, microenvironment, RNA sequencing, xenograft, porcine cancer model

## Abstract

Hepatocellular carcinoma (HCC) is the second leading cause of cancer-related death worldwide, representing the most common form of liver cancer. As HCC incidence and mortality continue to increase, there is a growing need for improved translational animal models to bridge the gap between basic HCC research and clinical practice to improve early detection and treatment strategies for this deadly disease. Recently the Oncopig cancer model—a novel transgenic swine model that recapitulates human cancer through Cre recombinase induced expression of *KRAS^*G*12*D*^* and *TP53^*R*167*H*^* driver mutations—has been validated as a large animal translational model for human HCC. Due to the similar size, anatomy, physiology, immunology, genetics, and epigenetics between pigs and humans, the Oncopig has the potential to improve translation of novel diagnostic and therapeutic modalities into clinical practice. Recent studies have demonstrated the importance of tumor cells in shaping its surrounding microenvironment into one that is more proliferative, invasive, and metastatic; however, little is known about the impact of microenvironment signaling on HCC tumor biology and differential gene expression between HCC tumors and its tumor microenvironment (TME). In this study, transcriptional profiling was performed on Oncopig HCC xenograft tumors (*n* = 3) produced via subcutaneous injection of Oncopig HCC cells into severe combined immunodeficiency (SCID) mice. To differentiate between gene expression in the tumor and surrounding tumor microenvironment, RNA-seq reads originating from porcine (HCC tumor) and murine (microenvironment) cells were bioinformatically separated using Xenome. Principle component analysis (PCA) demonstrated clustering by group based on the expression of orthologous genes. Genes contributing to each principal component were extracted and subjected to functional analysis to identify alterations in pathway signaling between HCC cells and the microenvironment. Altered expression of genes associated with hepatic fibrosis deposition, immune response, and neo angiogenesis were observed. The results of this study provide insights into the interplay between HCC and microenvironment signaling *in vivo*, improving our understanding of the interplay between HCC tumor cells, the surrounding tumor microenvironment, and the impact on HCC development and progression.

## Introduction

Solid tumors consists of a population of cancer cells in addition to a variety of resident and infiltrating host cells, secreted factors and extracellular matrix proteins, collectively known as the tumor microenvironment (TME). Crosstalk between tumor cells and the TME can lead to modulation of the TME, resulting in development of a beneficial microenvironment for tumor cells to grow and evade detection and killing by infiltrative immune cells (Whiteside, 2008).Tumor-derived signaling can not only allow tumor cells to escape the host immune system, but also promotes tumor cell growth. These interactions between cancer cells and the cellular and non-cellular components of the TME promote many aspects of tumor development, including cancer cell evolution, progression, and metastasis (Baghban et al., 2020). Understanding the underlying cellular and molecular mechanisms governing these interactions can inform development of novel therapeutic strategies designed to disrupt this signaling and restore anti-tumor activity (Whiteside, 2008).

Hepatocellular carcinoma (HCC) is the 5th most common cancer globally and the 2nd most common cause of cancer-related death worldwide, accounting for >9% of yearly cancer mortality (Flores and Marrero, 2014; Kirstein, 2016). HCC most commonly develops in cirrhotic liver microenvironments defined by increased fibrosis deposition and chronic inflammation, and there is growing evidence for the importance of cross-talk between tumor cells and the surrounding TME for modulating the progression of hepatocarcinogenesis, epithelial mesenchymal transitions, tumor invasion, and metastasis (Mhatre et al., 2012). HCC incidence in the United States has tripled over the past three decades due to the high incidence of liver cirrhosis from alcohol abuse and hepatitis C virus infection; the increasing prevalence of non-alcoholic steatohepatitis (NASH) due to the growing worldwide obesity epidemic ensures that HCC will continue to be an important public health concern in the future (Rahib et al., 2020). As HCC incidence and mortality continue to increase, there is a growing need for research into the impact of tumor cell-TME crosstalk to better understand HCC progression and develop novel treatment strategies for this deadly disease.

Advances in cancer research are reliant on the use of novel, clinically relevant preclinical cancer models. Recently the Oncopig Cancer Model—a novel transgenic swine model that recapitulates human cancer through Cre recombinase induced expression of *KRAS^*G*12*D*^* and *TP53^*R*167*H*^* driver mutations (Schook et al., 2015)—has been validated as a large animal translational model for human HCC (Schachtschneider et al., 2017a; Gaba et al., 2020). The similar size, anatomy, physiology, immunology, metabolism, genetics, and epigenetics between pigs and humans allows for translation of results to clinical practice to improve diagnostics and therapeutic applications (Schachtschneider et al., 2017b). This model can also provide insights into the molecular mechanisms involved in tumor development, signaling involved in development and maturation of the TME, and subsequent host responses towards cancer development.

The purpose of this study was to investigate HCC-TME crosstalk to better understand the HCC tumor cell and TME-derived mechanisms leading to development and modulation of the HCC TME. In order to distinguish between HCC tumor cell and TME signaling, RNA-seq was performed on Oncopig HCC xenograft tumors produced via injection of Oncopig HCC cells into SCID mice. To differentiate between gene expression in the tumor and surrounding tumor microenvironment, RNA-seq reads originating from porcine (tumor) and murine (TME) cells were separated bioinformatically to allow for individual quantification of HCC and TME-specific gene expression. The results of this study provide insights into the mechanisms through which HCC cells and the TME work in concert to modulate the TME and promote HCC proliferation.

## Methods

### Ethics Statement:

Animal studies and tissue collection was conducted at The University of Illinois at Urbana-Champaign in accordance with national and international guidelines and approved by The University of Illinois Institutional Animal Care and Use Committee (IACUC protocol numbers 10189 and 10163).

### Oncopig HCC Xenograft Induction and Collection:

Primary hepatocytes were isolated and transformed into HCC cells from Oncopig liver tissues (*n* = 2 female, *n* = 1 male) as previously described (Schachtschneider et al., 2017a; Gaba et al., 2020). Briefly, primary hepatocytes were isolated from resected Oncopig liver tissues. Twenty-four hours post isolation, primary hepatocytes were incubated with an adenoviral vector encoding Cre recominase, resulting in transgene expression and subsequent transformation into HCC cell lines. The 3 distinct Oncopig HCC cell lines were cultured for 3 weeks before injection into female SCID mice (NOD.CB17-Prkdcscid/JAX, Bar Harbor, ME, United States). For xenograft tumor generation, 1 × 10^7^ Oncopig HCC cells suspended in 100 μL phosphate buffered saline were subcutaneously injected into each mouse (*n* = 3). 21 days post injection, mice were euthanized, and tumors were harvested. Half of the tumor samples were flash frozen in liquid nitrogen and stored at −80 degrees Celsius until further processing. The other half was formalin fixed for histological confirmation of HCC.

### RNA Isolation:

Total RNA was extracted from the tumor samples using the AllPrep DNA/RNA Mini Kit (Qiagen, Valencia, CA, United States) following the manufacturer’s protocol. RNA integrity and confirmation of a lack of genomic DNA contamination was determined using an Agilent 2100 Bioanalyzer using an RNA Nano bioanalyzer chip by the Carver High-Throughput DNA Sequencing and Genotyping Unit (HTS lab, University of Illinois, Urbana, IL, United States). RNA samples were utilized for sequencing if their RNA integrity number was greater than 7.

### RNA-seq Library Preparation:

TruSeq Stranded RNA-seq libraries (TruSeq Stranded RNA Sample Preparation Kit, Illumina, San Diego, CA, United States) were produced from high-quality RNA (1 μg) by the HTS lab (University of Illinois, Urbana, IL, United States) following standard protocols. RNA-seq libraries were paired-end sequenced (2 × 100 bp) on an Illumina HiSeq2000. All datasets are available in the NCBI Short Read Archive under accession number PRJNA685801.

### Species-Based Read Separation and Mapping to Reference Genomes:

An average of 29 million raw stranded paired-end reads were produced for each sample, ranging from 28.4 to 29.4 million. Trim Galore version 0.4.4^1^ was used to trim raw reads for adapter contamination. Quality Phred score cutoff was set to 30, maximum trimming error rate was set to 0.1, and minimum sequence length was set to 20 with a stringency of 6. Reference genomes and transcriptomes were obtained from the Ensembl genome database. Mus_musculus.GRCm38.99 was used as the reference for mouse, and Sus_scrofa.Sscrofa11.1.99 (Warr et al., 2020) was used as the reference for pig. Xenome Version 1.0.0 from the Gossamer bioinformatics suite was used to separate reads between mouse and pig using default settings (Conway et al., 2012). Mouse was set as the host genome while pig was set as the graft genome. Reads were classified into the following classifications: host, graft, ambiguous, both, and neither. STAR 2.7.3a was used to map output reads from Xenome (Dobin et al., 2013). Only host and graft classified reads from Xenome were used for STAR alignment to the species-specific genome and transcriptome. The following non-default options were used for mapping with STAR: –sjdbOverhang 99, –outFilterType BySJout, –outFilterMultipmapNmax 20, –alignSJoverhangMin 8, –alignSJDBoverhangMin 1, –outFilterMismatchNmax 999, –outFilterMismatchNoverReadLmax 0.04, outSAMtype BAM SortedByCoordinate. A second pass mapping was performed using SJ.out.tab files from the first pass. RSEM version 1.3.1 was used to quantify gene expression (Li and Dewey, 2011). The following options were used with the rsem-calculate-expression function: –strandedness reverse, –alignments, –paired-end.

### Cross Species Analysis:

RStudio Version 1.2.5042 was used for data filtering and analysis of the RSEM gene quantifications. A list of known one-to-one orthologous genes between mouse and pig was created through Ensembl’s Biomart database. Genes were filtered to retain only one-to-one orthologues expressed in at least 1 sample. Transcripts per million (TPM) gene expression values were normalized through addition of a.01 pseudocount to all expression values followed by log base 2 transformation as previously described for comparison of gene expression across species (Foissac et al., 2019). Principle Component Analysis (PCA) plots were produced using the factoextra() package in RStudio Version 1.2.5042 using the prcomp() function. A list of genes that contributed to principle components 1 and 2 was extracted from the PCA. From these lists, all genes that were above the expected average contribution value were used for pathway analysis.

### Pathway Analysis:

The list of genes contributing to principle component 1 and 2 above the expected average contribution value were analyzed using Ingenuity Pathway Analysis (IPA) (QIAGEN Inc., Krämer et al., 2014)^2^. Average TPM values for each species were determined, and log2 fold changes were calculated between the average mouse and average pig TPM values. The following equation was used: Log2 fold change = Log2 (Average Mouse TPM/Average Pig TPM). Based on this calculation, a negative log2 fold change value indicates that the gene displays higher expression in the HCC tumor while a positive log 2 fold change value indicates that the gene displays higher expression in the Tumor Microenvironment (TME). Differentially expressed genes (DEGs) between the TME (murine genes) and the HCC tumor (porcine genes) were assigned as those that had log2 fold change values of ≤−2.2 or ≥ + 2.2. These values were selected to help ensure comparisons were based on biologically relevant differences in gene expression, as well as to allow for performance of pathway analysis using IPA’s recommended input range (200 to 3,500 genes). The principle component 1 and 2 gene lists alongside its corresponding log2 fold change value was uploaded for IPA’s core analysis (Supplementary Tables 1, 2). Core analysis revealed significantly enriched groups of genes associated with canonical pathways and diseases & functions derived from IPA’s database. *Z*-scores were calculated for each canonical pathway. The *Z*-scores reflect predicted activation or inhibition states of canonical pathways based off the expression of available genes in our dataset. With respect to our dataset, positive Z-scores indicate activation in the murine TME while negative *Z*-scores indicate activation in the porcine HCC tumors. *P*-values obtained from IPA are calculated using a Right-Tailed Fisher’s Exact Test. These *P*-values were then corrected for multiple testing using the Benjamin-Hochberg false discovery rate (FDR), using a FDR value of 0.05.

## Results

### Species-Based Read Separation:

Reads originating from mouse (TME) and pig (HCC) cells were successfully separated bioinformatically and assigned to their respective reference genomes (Table 1). The majority of reads (>93%) were either classified as Host (mouse, TME) or Graft (pig, HCC) in all samples. The average percentage of reads derived from the TME and HCC tumor cells was 52.2 and 42.3%, respectively (Table 1). Once separated, species-specific reads were aligned to their respective reference genomes (Supplementary Table 3). On average, mouse reads uniquely mapped to the mouse genome at a rate of 90.13%, while pig reads uniquely mapped to the pig genome at a rate of 86.08%. Given the ability to successfully separate and align reads based on species of origin, further analyses were performed to investigate HCC tumor and TME signaling patterns.

**TABLE 1:** Read Classifications.

	% “Host” Reads	% “Graft” Reads	% “Ambiguous” Reads	% “Both” Reads	% “Neither” Reads
Tumor 40	52.30%	42.60%	3.00%	0.77%	1.30%
Tumor 42	39.50%	54.10%	3.50%	0.97%	1.90%
Tumor 44	64.70%	30.20%	2.90%	0.88%	1.34%
Average	52.2%	42.3%	3.1%	0.87%	1.51%

*Proportion of reads Xenome classified to each category for all samples. The category “Host” refers to reads classified to the mouse reference genome while “Graft” refers to reads classified to the pig reference genome. The category “Both” refers to reads that match both mouse and pig reference genomes, “neither” represents reads that do not match either reference genome, and “Ambiguous” represents reads that are likely artifacts created during library preparation.*

### Genome-Wide Cross-Species Expression Analysis:

**FIGURE 1:**
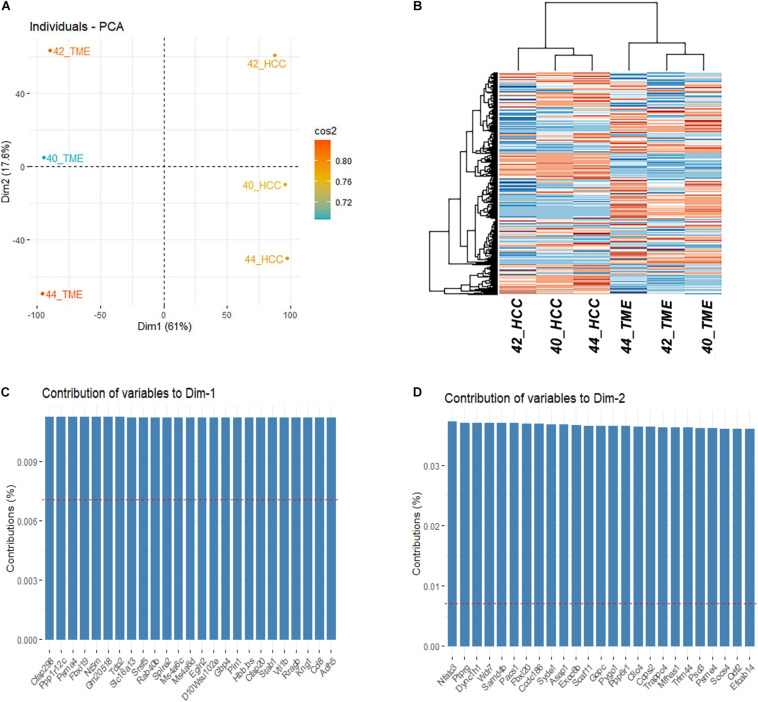
Differential Expression Between HCC Tumors and the TME. **(A)** PCA plot and **(B)** Heatmap based on the final filtered list of 14,163 orthologous genes expressed in at least 1 sample. **(C)** Top 25 genes contributing to the variation of dimension 1. **(D)** Top 25 genes contributing to the variation of dimension 2.

Gene expression levels were quantified for the 55,471 and 31,907 genes annotated in the mouse and pig genome, respectively. Of the 24,011 one-to-one orthologues identified between mice and pigs, 14,163 were expressed in at least one sample and used for downstream analyses. PCA and hierarchical clustering analysis based on the 14,163 one-to-one orthologues expressed in at least one sample resulted in samples clustering by group (Figures 1A,B). Principle component 1 represents 61% of the variation in the dataset and differentiates between the HCC tumor and TME samples. Interestingly, principle component 2, which represents 17.6% of the variation, differentiates between individual HCC tumor/TME sample pairs. In total 8,060 genes were identified to significantly contribute to principle component 1 (Figure 1C), while 4,603 genes significantly contributed to principle component 2 (Figure 1D).

### Pathway Enrichment Analysis:

In order to investigate the biological relevance of the differential gene expression observed between HCC tumor and TME samples, genes that contributed to each principle component at a higher rate than expected by chance were extracted for pathway analysis. As differential expression analysis was performed between HCC cells and subcutaneous TME cells (i.e., fibroblasts, inflammatory cells, vascular tissue), some of the differential expression observed may be due to differences in baseline expression between these cell and tissue types. Therefore, genes utilized for pathway analysis were further limited to those with a log2 fold change in expression of ≤−2.2 or ≥+2.2 to help ensure comparisons were based on biologically relevant differences in gene expression while reducing noise related to baseline expression differences between cell types. The final number of DEGs used for pathway analysis was 3,347 for principal component 1 (1,424 genes upregulated in the HCC tumor and 1,923 genes upregulated in the TME) and 269 genes for component 2 (114 genes upregulated in the HCC tumor and 155 genes upregulated in the TME). Functional enrichment analysis of principal component 1 genes resulted in identification of pathways involved in hepatic fibrosis, immune system regulation, inflammation regulation, and neo angiogenesis (Table 2). In addition, the top diseases and disorders enriched for genes from principal component 1 included cancer, organismal injury and abnormalities, and gastrointestinal disease (Supplementary Table 4), while the top enriched molecular and cellular functions included cellular movement and cell-to-cell signaling interaction (Supplementary Table 5). Finally, the top physiological system development and functions enriched for genes from principal component 1 included immune cell trafficking (Supplementary Table 6). Pathway analysis on principle component 2 did not reveal enrichment of any canonical pathways. However, top diseases and disorders enriched for genes from principal component 2 included cancer, endocrine system disorders, and gastrointestinal disease (Supplementary Table 7).

**TABLE 2:** Top 20 Pathways enriched for genes significantly contributing to principal component 1.

Ingenuity Canonical Pathways	adjusted *p*-value	Ratio	z-score	Total genes	# of genes upregulated in HCC tumor	# of genes upregualted in TME
1. Hepatic Fibrosis/Hepatic Stellate Cell Activation	4.11E-13	0.371	undetermined	69	16	53
2. Axonal Guidance Signaling	1.3E-11	0.264	undetermined	128	50	78
3. Leukocyte Extravasation Signaling	4.33E-11	0.34	1.192	67	26	41
4. Atherosclerosis Signaling	2.05E-10	0.386	undetermined	49	21	28
5. Granulocyte Adhesion and Diapedesis	2.73E-10	0.339	undetermined	61	30	31
6. LXR/RXR Activation	2.73E-10	0.388	−3.618	47	27	20
7. FXR/RXR Activation	5E–09	0.365	undetermined	46	36	8
8. Coagulation System	7.78E–09	0.6	−0.218	21	16	5
9. Agranulocyte Adhesion and Diapedesis	1.35E–08	0.311	undetermined	43	20	23
10. GP6 Signaling Pathway	1.83E–08	0.361	4.727	43	7	36
11. Breast Cancer Regulation by Stathmin1	1.83E–08	0.23	undetermined	136	48	88
12. Role of Macrophages, Fibroblasts and Endothelial Cells in Rheumatoid Arthritis	7.85E–08	0.263	undetermined	82	26	56
13. Cardiac Hypertrophy Signaling (Enhanced)	2.51E–07	0.232	2.83	113	44	69
14. Role of Osteoblasts, Osteoclasts and Chondrocytes in Rheumatoid Arthritis	7.72E–07	0.277	undetermined	61	25	36
15. Sperm Motility	1.22E–06	0.274	2.2	61	21	40
16. Role of Pattern Recognition Receptors in Recognition of Bacteria and Viruses	6.97E–06	0.292	2.6	45	13	32
17. Hepatic Fibrosis Signaling Pathway	6.97E–06	0.234	2.871	82	29	53
18. Calcium Signaling	9.84E–06	0.267	2.271	55	17	38
19. Colorectal Cancer Metastasis Signaling	9.84E–06	0.253	2.405	64	25	39
20. Synaptogenesis Signaling Pathway	9.84E–06	0.24	1.65	75	31	44

*Top 20 pathways enriched for genes significantly contributing to principal component 1 with adjusted *p*-values derived from the Benjamini-Hochberg procedure. The ratio represents number of genes that maps to the pathway divided by the total number of genes in the pathway. The *z*-scores provide information regarding activation status; *z*-scores >2 predict activation of the pathway in the TME while z-scores <−2 predicts activation of the pathway in the HCC tumor.*

### Hepatic Fibrosis Induced by Signaling Between HCC Tumor and Its Microenvironment:

**FIGURE 2:**
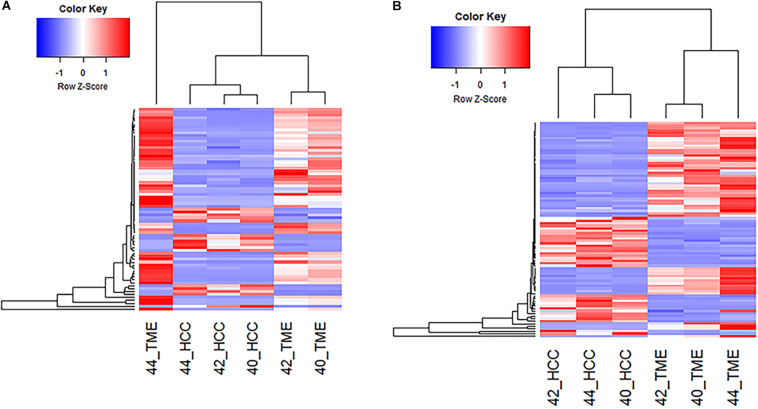
Differential Expression of Genes Involved in Hepatic Fibrosis. **(A)** Heatmap of the 69 genes differentially expressed in the Hepatic Stellate Cell Activation Pathway for all 6 samples. **(B)** Heatmap of the 82 differentially expressed genes in the Hepatic Fibrosis Signaling Pathway for all 6 samples.

Pathway analysis revealed enrichment of two liver specific pathways involved in fibrosis: hepatic stellate cell (HSC) activation pathway and hepatic fibrosis signaling pathway, with the HSC activation pathway representing the most significantly enriched pathway (−log(*p*-value) = 14.7; Table 2). The hepatic fibrosis signaling pathway had a *Z*-score greater than 2 indicating that this pathway is significantly upregulated in the TME. A total of 69 genes in the HSC Activation Pathway were differentially expressed in this study, 53 and 16 of which displayed higher expression in the TME and HCC tumors, respectively (Figure 2A). In addition, a total of 82 genes in the Hepatic Fibrosis Signaling pathway were altered in this study, 52 and 29 of which displayed higher expression in the TME and HCC tumors, respectively (Figure 2B).

**FIGURE 3:**
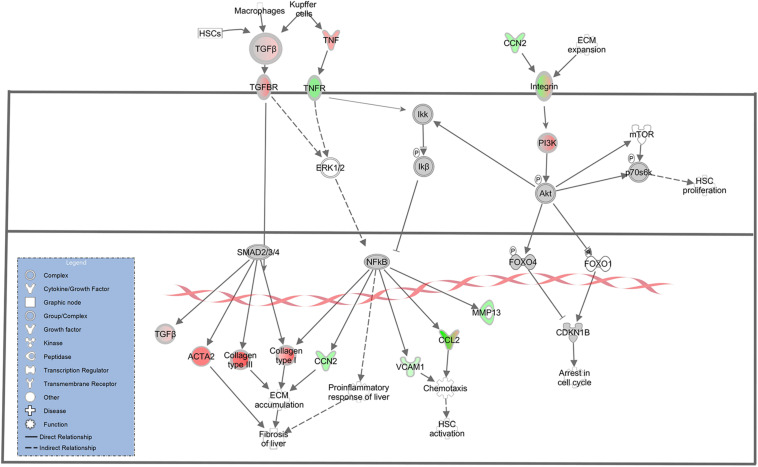
Differential Expression in the Hepatic Fibrosis Signaling Pathway. Differential expression of genes in the hepatic fibrosis signaling canonical pathway in IPA (edited for simpler visualization; for full pathway, see Supplementary Figure 1). Molecule interactions are shown as explained in the legend. DEGs are highlighted either in red (upregulated TME genes) or green (upregulated HCC tumor genes). Color intensity indicates Increasing or decreasing degree of fold change.

A key molecular and cellular driver in the progression of liver fibrosis is the activation of HSCs. In normal physiological liver function, quiescent HSCs act as a storage for Vitamin A. Expression of inflammatory molecules and mediators following acute liver injury causes HSC activation and transdifferentiation into highly proliferative, pro-inflammatory, and migratory myofibroblasts that produce collagen and many other extracellular matrix (ECM) proteins (Puche et al., 2013; Zhang et al., 2016; Higashi et al., 2017). In this study, myofibroblast activation was observed as evidenced by increased *ACTA2* in the TME (log2 fold change = 6.52). *ACTA2* is a proven myofibroblast marker, and is highly expressed in myofibroblasts irrespective of their precursor cell type (Watsky et al., 2010). The presence of myofibroblasts in the TME was likely driven by increased expression of *TGFB* (log2 fold change = 2.78) and its receptor *TGFBR3* (log2 fold change = 6.29) in the TME, as *TGFB* is one of the most potent activators of myofibroblast differentiation and has been shown to induce *ACTA2* expression (Harris et al., 2013; Breton et al., 2018). Additionally, IPA revealed *ACTA2* upregulation via *TGFB* mediated regulation (Figure 3). While these results demonstrate that TME cells represent the source of *TGFB* secretion, *TGFB* must be activated from its latent form in order to induce myofibroblast differentiation. Key *TGFB* activators include integrin proteins (Goodwin and Jenkins, 2009; Khan and Marshall, 2016), 4 of which (*ITGA2, ITGA3, ITGB1, ITGB3*) displayed increased expression in the HCC tumor cells (Log2 fold change = −6.16, −5.56, −2.32, and −4.35, respectively). Additionally, IPA revealed an interaction between *CCN2* and integrins that could lead to inhibition of cell cycle arrest and HSC proliferation through *AKT* signaling (Figure 3). Increased *CCN2* expression (log2 fold change = −4.10) was observed in the HCC tumor cells and is associated with *TGFB*-mediated fibrosis deposition (Nakerakanti et al., 2011).

**FIGURE 4:**
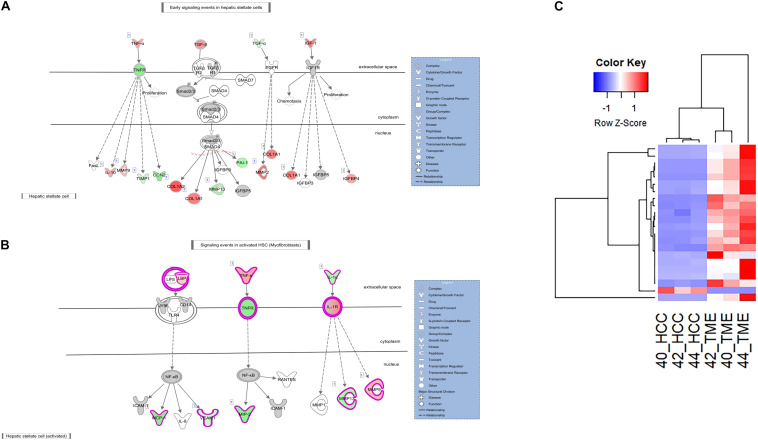
Differential Expression in the Hepatic Stellate Cell Activation Pathway. **(A,B)** The hepatic stellate cell activation canonical pathway in IPA (edited for simpler visualization; for full pathway, see Supplementary Figure 2). Molecule interactions are shown as explained in the legend. DEGS are highlighted either in red (upregulated TME genes) or green (upregulated HCC tumor genes). Color intensity indicates increasing or decreasing degree of fold change. **(C)** Heatmap of the 22 collagen genes differentially expressed in the Hepatic Stellate Cell Activation Pathway for all 6 samples.

In addition to increased *ACTA2* expression, 21 type I and type III collagen genes showed increased expression in the TME (Figures 3, 4). Only one collagen gene, *COL17A1* (log2 fold change = −8.43), was found to be upregulated in the HCC tumor. In addition, increased expression of genes promoting collagen production was observed in both the HCC tumor and TME cells. In the TME, increased *TGFB* expression promotes type I and type III collagen production (Figures 3, 4), while increased *IGF1* expression (log2 fold change = 6.55) promotes production of type 1 collagen (Figure 4). In addition, increased expression of *TGFA* (log2 fold change = −2.28), a potent mitogen for hepatocytes that is associated with hepatocarcinogenesis (Harada et al., 1999; Yeh et al., 2007), was observed in HCC cells resulting in upregulation of type I collagen (Figure 4).

ECM remodeling proteins were also differentially expressed in this study. Matrix metalloproteinases (MMPs) and tissue inhibitors of metalloproteinases (TIMPs) are major players in ECM remodeling and play a role in HCC progression and invasion (Scheau et al., 2019). In this study, multiple MMPs and TIMPs displayed altered expression between the HCC tumor and TME. *MMP2* (log2 fold change = 7.47), *MMP9* (log2 fold change = 3.63), and *TIMP2* (log2 fold change = 2.66) displayed increased expression in the TME, while *MMP13* (log2 fold change = −4.41) and *TIMP1* (log2 fold change = −2.26) displayed increased expression in the HCC tumor. In addition, increased expression of several genes in the TME known to drive expression of HCC tumor-derived *TIMP1* and *MMP13* was observed (Figures 3, 4). These factors include *TGFB* and *TNF* (log2 fold change = 2.78 and 4.38, respectively). IPA revealed *TGFB* mediated upregulation of *MMP13* (Figure 4A) and *TNF* mediated upregulation of *MMP13* (Figure 3). Likewise, increased expression of *IL1A* (log2 fold change = −4.15) and *TGFA* (log2 fold change = −2.28) was observed in the HCC tumor cells leading to increased expression of TME-derived *MMP9* and *MMP2*, respectively (Figure 4).

### Innate Immune System and Inflammatory Signals Derived From HCC Cells:

**FIGURE 5:**
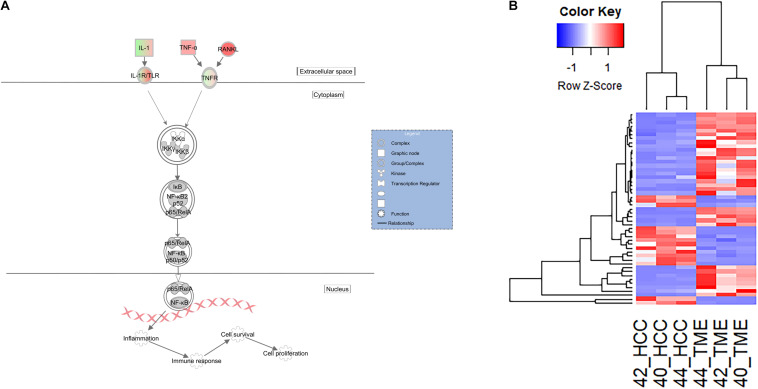
Differential Expression of Genes Inolved in the Innate Immune and Inflammatory System. **(A)** Differential expression within the NF-κB canonical pathway in IPA (edited for simpler visualization; for full pathway, see Supplementary Figure 3). Molecule interactions are shown as explained in the legend. DEGs are highlighted either in red (upregulated TME genes) or green (upregulated HCC tumor genes). Color intensity indicates Increasing or decreasing degree of fold change. **(B)** Heatmap of the 49 genes differentially expressed in the NF-κB signaling pathway for all 6 samples.

In our dataset, pathway analysis revealed enrichment of multiple pathways associated with innate immune surveillance and chronic inflammation (Table 2). These pathways include the previously described hepatic fibrosis pathways, and NF-κB signaling (−log(*p*-value) = 5.81, *Z*-score = 2.598). The NF-κB signaling pathway is activated in the TME and contains 49 DEGs, 35 of which displayed higher expression in the TME and 14 with higher expression in the HCC tumors (Figure 5).

With respect to the hepatic fibrosis pathways, DEGs of interest include *TNF, TNFRSF1B*, and *LBP*. *TNF* displayed higher expression in the TME (log2 fold change = 4.38), while its binding receptor *TNFRSF1B* displayed higher expression in the HCC tumor (log2 fold change = −2.25). *TNF* is a major inflammatory cytokine produced mainly by macrophages and is a key contributor to many malignancies including HCC (Liu et al., 2013; Tan et al., 2019). In our dataset, TME-derived *TNF* appears to interact with HCC tumor cells expressing *TNFRSF1B* to induce upregulation of HCC-derived *CCN2* (log2 fold change = −4.10), *MIP2* (log2 fold change = −6.14), and *VCAM1* (log2 fold change = −2.46; Figures 3, 4A). *CCN2* is a secreted growth factor that interacts with various ECM molecules and is known to promote HCC progression through increased proliferation of cancer-associated fibroblasts and increased migration of macrophages (Mazzocca et al., 2010; Akahoshi et al., 2016; Makino et al., 2018). *MIP2* is a cytokine that recruits neutrophils in the setting of liver injury (Moles et al., 2014; Qin et al., 2017), while *VCAM1* expression during inflammatory diseases provides a scaffold for leukocyte migration and infiltration (Cook-Mills et al., 2011).

In the pathogenesis of liver injury, lipopolysaccharides from bacteria play a role as potent mediators of hepatic inflammation and fibrosis. *LBP* encodes a protein that binds to bacterial lipopolysaccharides, eliciting an immune response (Gandhi, 2020). In our dataset, *LBP* (log2 fold change = 2.67) displayed higher expression in the TME. Myofibroblasts bind to LBP-lipopolysaccharide complexes to induce expression of HCC tumor expressed *MCP1* (log2 fold change = −7.42) *and VCAM1* (log2 fold change = −2.46; Figure 4B), which displayed increased expression in the HCC tumors in this study. *MCP1* is an inflammatory chemokine that is crucial for monocyte infiltration into tissues during inflammation (Germain, 2017). *VCAM1* is a protein capable of mediating adhesion of lymphocytes to the vascular endothelium which include increased positive expression in inflamed liver sinusoids (Osborn et al., 1989; Steinhoff et al., 1993; Ho et al., 2004).

NF-κB is a key transcriptional regulator of inflammatory responses and plays a prominent role in chronic liver diseases (Luedde and Schwabe, 2011). In this study, the NF-κB signaling pathway was significantly activated in the TME (*Z*-score = 2.6). Activation of NF-κB leads to expression of pro-inflammatory genes and regulates the survival and activation of innate immune cells (Liu et al., 2017). DEGs identified in this pathway include *IL1A*, *TNF*, *TGFA*, and *RANKL*. *RANKL* (log2 fold change = 7.39) is a potent stimulator of NF-κB and its expression is associated with tumor migration and invasion in HCC (Song et al., 2014). It is upregulated in the TME and may induce NF-κB signaling via binding to one of the HCC tumor expressed receptors, such as *TNFRSF1B* (Figure 5; log2 fold change = −2.24). *IL1A* (log2 fold change = −4.15) is upregulated in the HCC tumors and is likely signaling through *IL1R2* (Log2 fold change = 4.74) which is expressed in the TME. *IL1R2* is a decoy receptor that IL1A can bind in order to prevent IL1A mediated signal transduction and may play protective roles in chronic inflammatory diseases like ulcerative colitis (Mora-Buch et al., 2016) and liver cirrhosis. This suggests that the TME could be responding to chemokines being produced by HCC tumors in order to limit an overactive inflammatory and immune response. As a consequence, the HCC tumors would be primed to grow in the immunotolerant microenvironment.

### Neo Angiogenesis Induced by HCC Tumor Signaling:

**FIGURE 6:**
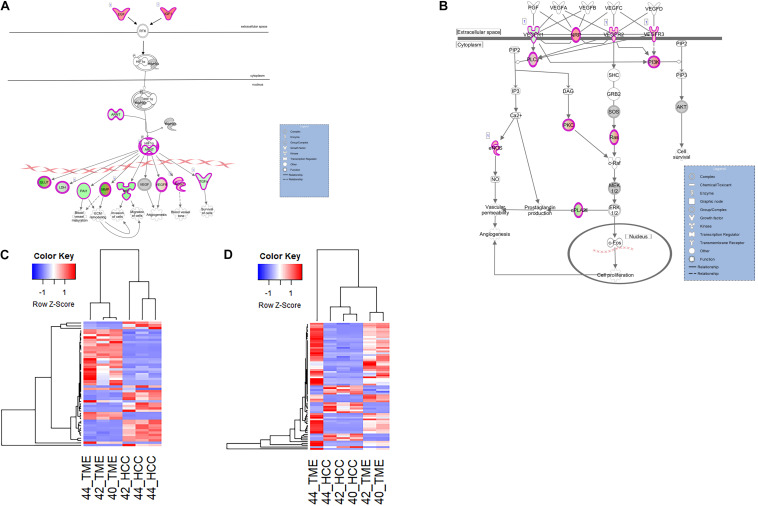
Differential Expression of Genes Involved in Neo angiogenesis. Differential expression within the **(A)** HIF1α Signaling canonical pathway and **(B)** VEGF Family Ligand-Receptor Interactions canonical pathway in IPA. Both pathways are edited for simpler visualization (for full pathway, see Supplementary Figures 4, 5). Molecule interactions are shown as explained in the legend. DEGs are highlighted either in red (upregulated TME genes) or green (upregulated HCC tumor genes). Color intensity indicates increasing or decreasing degree of fold change. **(C)** Heatmap of the 51 DEGs in the HIF1α Signaling pathway for all 6 samples. **(D)** Heatmap of the 20 DEGs in the VEGF Family Ligand-Receptor Interactions pathway for all 6 samples.

In this study, two pathways related to neo angiogenesis were enriched for DEGs: the HIF1α signaling pathway and the VEGF family ligand-receptor interactions pathway. The HIF1α signaling pathway was activated in the TME (−log(*p*-value) = 4.74; *Z*-score = 2.1) and contains 51 DEGs, 31 and 20 of which display higher expression in the TME and HCC tumors, respectively (Figure 6). The VEGF Family Ligand-Receptor Interactions pathway (−log(*p*-value) = 2; *Z*-score = 1.41) is likely activated in the TME and contains 20 DEGs, 14 and 6 of which display higher expression in the TME and HCC tumors, respectively (Figure 6).

Hif1α is an important promoter of angiogenesis during hypoxic conditions. As HCC tumors are one of the most hypoxic tumors (Chen and Lou, 2017), Hif1α signaling is likely to be involved in HCC neovascularization. In our dataset, DEGs in the HIF1α signaling pathway and VEGF family ligand-receptor interactions pathway that contribute to neo angiogenic growth include various vascular endothelial growth factors (VEGFRs), *IGF1*, *EGF, ARNT*, *PAI1, and TGFA*. Pathway analysis revealed two TME-derived growth factors, *IGF1* (log2 fold change = 6.55) and *EGF* (log2 fold change = 5.24) that are driving Hif1α signaling within the HCC tumor cells (Figure 6). This is evidenced by increased expression of HCC tumor-derived genes downstream of the Hif1α-*ARNT* complex (Figure 6). HCC tumor-derived *ARNT* (log2 fold change = −2.28) is a crucial gene involved in tumor progression that binds to Hif1α during hypoxic conditions to upregulate genes that contribute to invasion, metastasis, and neo angiogenesis (Tanimoto, 2000; Weir et al., 2011). From the data, the Hif1α-ARNT complex induces expression of proangiogenic factors including VEGFRs, *PAI1* (log2 fold change = −4.08), and *TGFA* (log2 fold change = −2.27). Additionally in the VEGF family ligand-receptor interactions pathway, *VEGFR1* (log2 fold change = −2.38) was upregulated in the HCC tumor while *NRP1* (log2 fold change = 4.93), *VEGFR2* (log2 fold change = 2.76), and *VEGFR3* (log2 fold change = 3.21) were upregulated in the TME.

## Discussion

In this study, we sought to examine differential gene expression between HCC tumor cells and TME cells by looking at genes expressed between porcine HCC tumor cells xenografted into SCID mice via RNA-seq and pathway analysis with IPA. Separating porcine HCC tumor and murine TME RNA-seq reads revealed a greater understanding of signaling pathway alterations and cross-talk between HCC tumor cells and its TME. The main signaling interactions observed between HCC tumors and their TME were related to hepatic fibrosis, inflammation, and neo angiogenesis mechanisms.

HCC typically develops in cirrhotic livers following hepatic fibrosis deposition; however, few studies have investigated how interactions between HCC tumor cells and TME cells mediate changes in hepatic fibrosis during HCC progression. In this study, two pathways related to hepatic fibrosis signaling were enriched for DEGs: The HSC Activation Pathway and The Hepatic Fibrosis Signaling Pathway. A key molecular and cellular driver in the progression of liver fibrosis is the activation of HSCs. In this study, myofibroblast activation was observed as evidenced by increased *ACTA2* in the TME. The increased expression of TME-derived *ACTA2* is likely due to increased *TGFB* expression in the TME, which is activated from its latent form by integrin proteins. Interestingly, several integrins were upregulated in the HCC tumors, suggesting that HCC tumor cells are actively promoting *TGFB*-mediated activation of myofibroblasts. In addition to increased *ACTA2* expression, the presence of activated myofibroblasts is further evidenced by upregulation of 21 type I and type III collagen genes in the TME. Type I and type III collagen represent the most abundant types of collagen found in fibrotic/cirrhotic liver tissues (Rojkind et al., 1979; Aycock and Seyer, 2009; Decaris et al., 2015), and activated myofibroblasts are known to be one of the greatest producers of these collagen types during liver fibrosis deposition (Shang et al., 2018). Together, the combined expression of TME-derived (*TGFB* and *IGF1*) and HCC-tumor derived (*TGFA*) upstream regulators of HSC activation suggests both the HCC tumor cells and TME cells are promoting collagen deposition.

In addition to evidence of collagen deposition, differential expression of genes involved in ECM remodeling was also observed. MMPs and TIMPs are major players in ECM remodeling and play a role in HCC progression and invasion (Scheau et al., 2019). In this study, overexpression of *MMP13* and *TIMP1* was observed in the HCC tumor cells. The increased expression was linked to TME-derived *TGFB* and *TNF*, as both *TGFB* and *TNF* are known to be potent inducers of *MMP13* (Leivonen et al., 2002; Liacini et al., 2003), while *TNF* is known to modulate *TIMP1* expression (Li et al., 1999; Nee et al., 2004). Additionally, HCC derived *IL1A* and *TGFA* appear to be promoting upregulation of TME-derived *MMP9 and MMP2*, respectively. *TIMP2* and *TIMP1* are specific inhibitors of *MMP2* and *MMP9*, respectively (Chen et al., 2020). *MMP2* degrades ECM components of the basement membrane and facilitates tumor invasion while *TIMP2* regulates *MMP2* activity. In the context of clinical observations in HCC patients, several studies have revealed that an imbalance of *MMP2* and *TIMP2* expression is associated with poorer prognosis of HCC (Giannelli et al., 2002; Daniele et al., 2014). As both *MMP2* and *TIMP2* are expressed at high levels in the TME of this study, these results suggest that the clinically observed imbalance in *MMP2* and *TIMP2* expression is likely mediated by the TME as opposed to the HCC cells themselves. Like *MMP2*, *MMP9* also degrades ECM components of the basement membrane to help facilitate tumor invasion while *TIMP1* regulates *MMP9* activity. Clinical observations have shown that higher expression of *MMP9* is associated with capsular HCC infiltration (Arii et al., 1996). The increased *MMP9* expression observed in the TME of this study suggests this clinical observation is also mediated by the TME as opposed to the HCC cells, although further studies are required to confirm these results.

In addition to hepatic fibrosis induction via myofibroblast activation and ECM remodeling, inflammation plays an essential role in mediating hepatic fibrosis and HCC progression. It has been extensively shown that chronic liver inflammation is associated with fibrosis and progression of HCC (Marra and Tacke, 2014; Tsuchida and Friedman, 2017; Ringelhan et al., 2018). In this study, evidence of TME-derived *TNF* binding to its receptor *TNFRSF1B* on HCC tumor cells was observed, resulting in upregulation of HCC-derived *CCN2, MIP2, and VCAM1.* These three genes play a role in recruiting macrophages and other leukocytes to sites of inflammation. In addition, *LBP* derived from the TME was also found to promote expression of HCC-derived *MCP1* and *VCAM1*, both of which are involved in monocyte recruitment. This suggests that the HCC tumor cells are producing the necessary factors to promote local inflammation rather than TME cells responding to the abnormal cell growth. However, as this study was performed in SCID mice with compromised immune responses, the lack of signaling in the TME could also be due to the weakened immune system in this model. Therefore, further studies using immunocompetent model systems are required to further investigate the role of the TME in leukocyte recruitment. In addition to the hepatic fibrosis pathway, the NF-κB signaling pathway was also significantly activated in the TME in this study. NF-κB is a key transcriptional regulator of inflammatory responses and plays a prominent role in chronic liver diseases (Luedde and Schwabe, 2011). Although this pathway was significantly activated in the TME, differential expression of NF-κB target genes was not observed. This is likely due to binding of HCC-derived *IL1A* to TME-derived *IL1R2*, a decoy receptor that binds IL1A to prevent subsequent signal transduction. This result suggests that the TME is expressing *IL1R2* in order to limit an overactive inflammatory and immune response, providing an immunotolerant microenvironment for the HCC tumors.

HCC tumors in general are characterized as highly vascularized and hypoxic tumors (Yang and Poon, 2008; Chen and Lou, 2017). However, the mechanisms through which HCC tumors become vascularized and hypoxic are not well understood. In this study, two pathways related to angiogenesis were enriched for DEGs and activated in the TME: the HIF1α signaling pathway and the VEGF family ligand-receptor interactions pathway. In the Hif1α signaling pathway, TME-derived growth factors *IGF* and *EGF* are likely promoting HCC-tumor derived *ARNT*, which is in turn promoting the expression of known pro-angiogenesis genes *PAI1, and TGFA*. Studies have shown that *PAI1* is upregulated in several cancers and represents a major contributor to angiogenesis in many cancers including HCC (Isogai et al., 2001; Geis et al., 2015). As described above, *TGFA* was upregulated in the HCC tumors acting as an upstream regulator of hepatic fibrosis. *TGFA* is also a pro-angiogenic factor whose expression is induced via activation of the HIF1α signaling pathway, and has been shown to be a potent angiogenic factor in a variety of tumors (Schreiber et al., 1986). Although studies have shown overexpression of *TGFA* in HCC preclinical models and in clinical studies (Tomiya and Fujiwara, 1996; Jaiswal, 2014), there is limited knowledge on the angiogenic effect of *TGFA* specifically in HCC tumors. Our data suggests that *TGFA* could be stimulating HCC growth and fibrosis via its angiogenic potential.

With regards to the VEGF family ligand-receptor interactions pathway, *VEGFR1* and *VEGFR2* are high-affinity receptors for angiogenesis factors and have been found to play key autocrine signaling roles in HCC cell proliferation (de Vries et al., 1992; Quinn et al., 1993; Peng et al., 2014). When bound to their ligands, predominantly *VEGF*, downstream signaling leads to promoting vascular permeability and endothelial cell proliferation. *VEGFR1* plays critical roles in promoting pathological tumor angiogenesis and invasion (Ceci et al., 2020), and HCC tumor cells have been shown to express *VEGFR1* resulting in induction of autocrine VEGF signaling (Peng et al., 2014). *VEGFR2* expression regulates vascular endothelial cell function by promoting proliferation and survival (Holmes et al., 2007), while *NRP1* is a pro-angiogenic co-receptor and has been shown to be expressed in both HCC tumor cells and hepatic endothelial cells (Bergé et al., 2011). Therefore, TME-derived *VEGFR2* and *NRP1* is likely being expressed by endothelial cells surrounding the HCC tumor in order to promote a new vascular network for the xenograft. As differential expression of ligands that bind to any of the VEGFRs was not observed, it is uncertain whether VEGF signaling is actually occurring at this point in the tumorigenic process, although previous studies have demonstrated the hypervascular nature of Oncopig HCC xenografts (Schachtschneider et al., 2017a). However, HCC tumor and TME-derived expression of several VEGFRs demonstrates that the HCC tumor and TME cells are displaying critical receptors for promotion of angiogenesis.

In conclusion, this study investigated gene expression profiles in the Oncopig HCC tumor xenograft model, identifying crosstalk between HCC tumors and the TME related to HCC progression, including hepatic fibrosis, inflammation, and angiogenesis. We revealed multiple pathways and specific gene interactions between HCC tumors and the TME that provide insights into how HCC tumor and TME cells cooperate to promote HCC proliferation. However, several limitations were identified in this study. First, the small sample size used limits our ability to control for variability and identify outliers, and therefore larger further confirmatory studies are required. The small sample size also limited our ability to investigate the possible gender-based stratification of samples observed on principal component 2. Second, although TP53 driver mutations are commonly observed in clinical HCC, the KRAS driver mutation is rarely observed in HCC (Hussain et al., 2007; Turhal et al., 2015). However, previous publications have demonstrated that Oncopig HCC recapitulates human HCC in terms of transcriptional profiles, histological phenotypes, AFP expression, proliferation rates, migration rates, and chemotherapeutic susceptibilities (Schachtschneider et al., 2017a; Gaba et al., 2020). Therefore, despite the presence of a KRAS driver mutation, the Oncopig HCC model represents a clinically relevant large animal HCC model. Additionally, while utilizing RNA-seq data allows for genome-wide profiling of all expressed genes, the cross-species analysis performed in this study limited the analysis to one-to-one orthologues. As the final one-to-one orthologue set used in this study represents 25.5% and 44.4% of genes annotated in the mouse and pig genome, respectively, our ability to fully elucidate all the pathway disruptions and HCC-TME crosstalk is limited. In addition, as RNA-seq only allows for quantification of gene expression at the mRNA level, further studies are required to investigate the impact of protein-protein interactions and post-translational modifications on HCC tumor development, as well as confirm the results of the pathway analysis presented here. Finally, as the presence of tumor infiltrating lymphocytes and other immune cells is a key component of the TME, our inability to fully characterize signaling originating from these cell types represents a limitation of this and other studies using SCID mice for xenograft development. Therefore, while this model allows for efficient bioinformatics-based read separation to assess HCC and TME cross talk, elucidation of the role the immune system plays could not be determined.

## Data Availability Statement

The datasets presented in this study can be found in online repositories. The names of the repository/repositories and accession number(s) can be found below: https://www.ncbi.nlm.nih.gov/sra, PRJNA685801.

## Ethics Statement

The animal study was reviewed and approved by University of Illinois at Urbana-Champaign IACUC.

## Author Contributions

KS, ME-K, LS, and RG conceptualized and designed the study. SP, KS, and AS performed the experiments. SP and AS performed the data analysis. SP wrote the manuscript. All authors contributed to the discussion of results and manuscript corrections.

## Conflict of Interest

RG receives research funding from the National Insitutes of Health, the United States Department of Defense, Guerbet United States LLC, and Janssen Research Development LLC. KS receives research funding from the National Insitutes of Health, Guerbet United States LLC, and Janssen Research Development LLC. RG, KS, and LS are scientific consultants for Sus Clinicals, Inc.

## Footnotes

1 https://github.com/FelixKrueger/TrimGalore

2 https://www.qiagenbioinformatics.com/products/ingenuitypathway-analysis
