# Supplementary material for: Transcriptional Profiling of Porcine HCC Xenografts Provides Insights Into Tumor Cell Microenvironment Signaling
Source: Front Genet. 2021 Apr 29;12:657330. doi: 10.3389/fgene.2021.657330 (PMC8118521; doi:10.3389/fgene.2021.657330)
Supplement: Supplementary file 1 [file Data_Sheet_1.docx]

**Supplemental Figure 1. Hepatic Fibrosis Signaling Pathway**


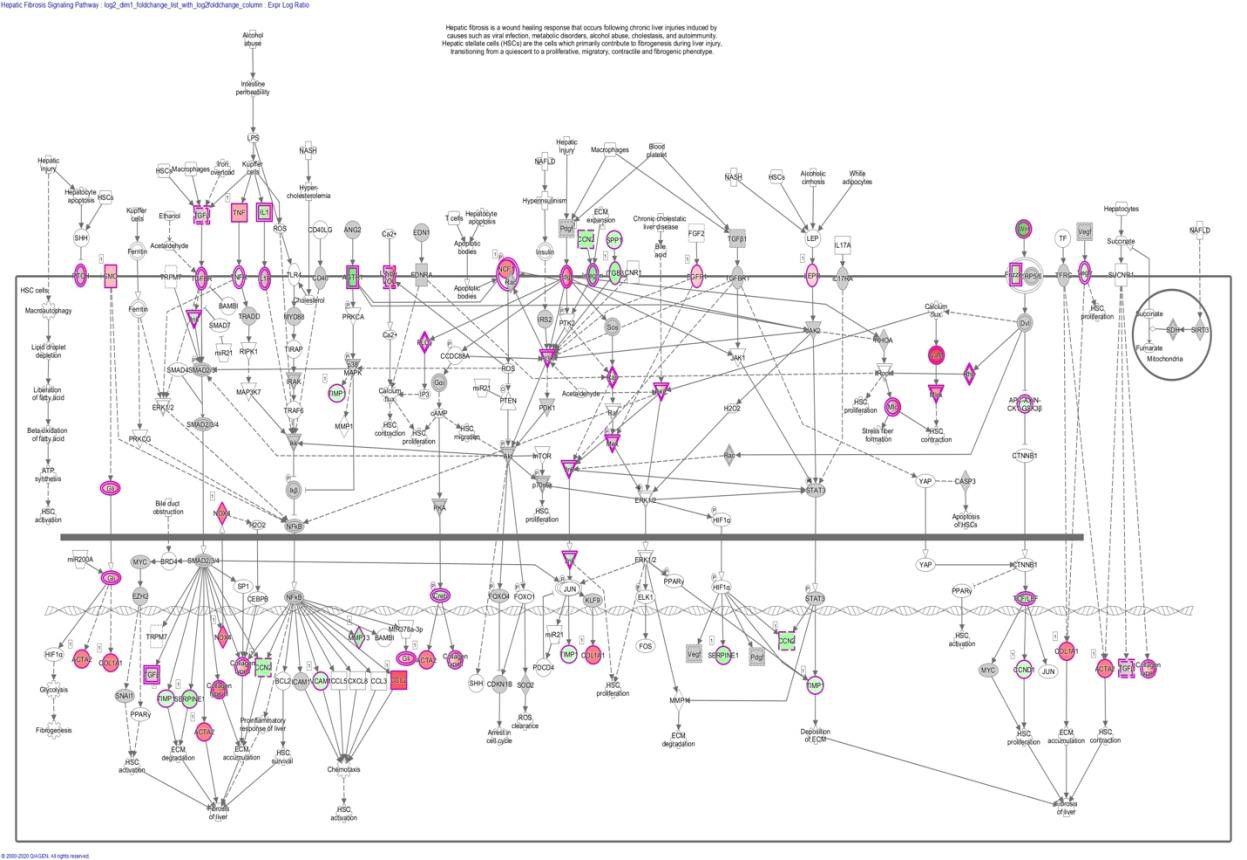


**Supplemental Figure 2. Hepatic Stellate Cell Activation pathway**


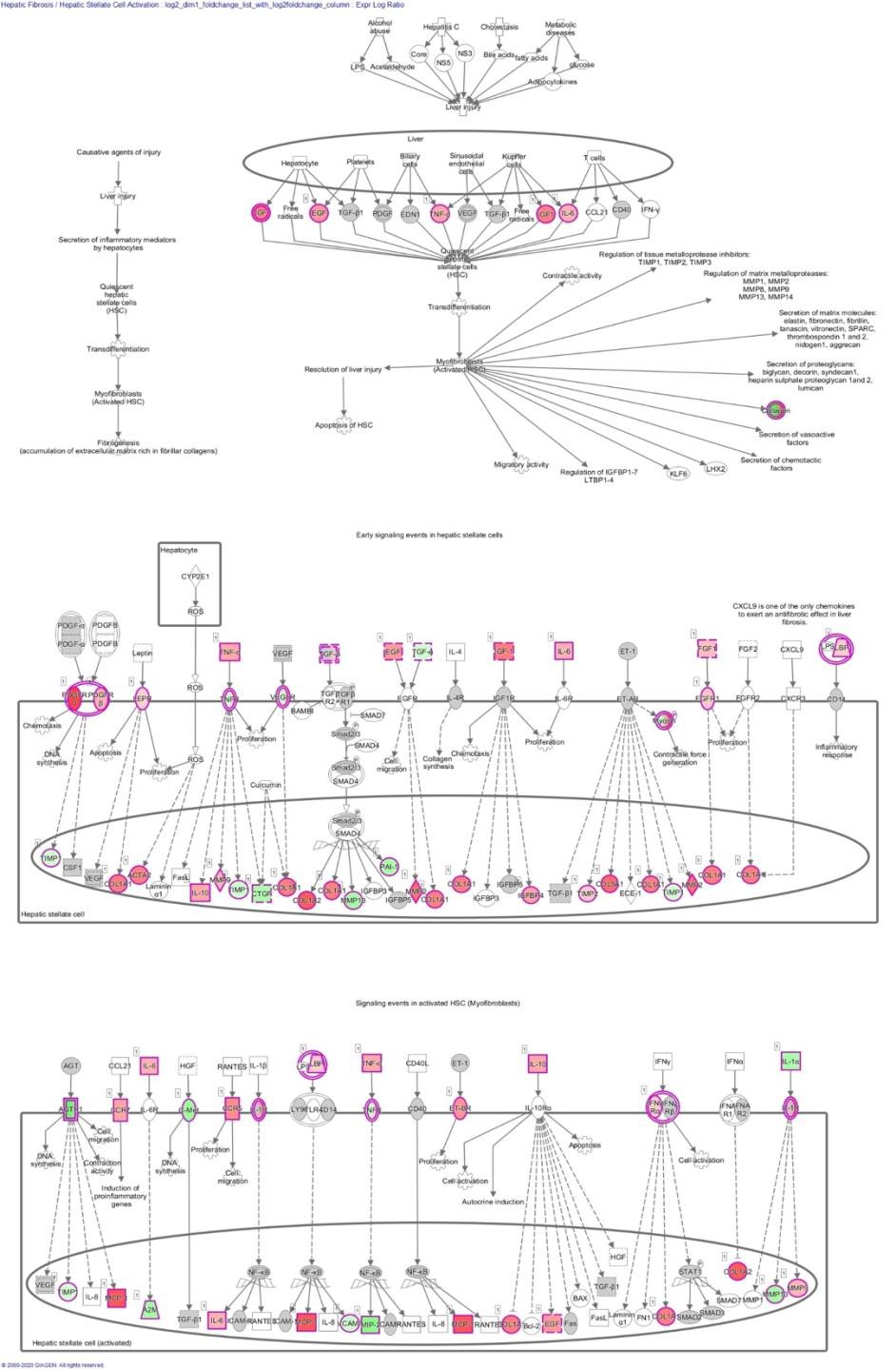


**Supplemental Figure 3. NF-kB Signaling Pathway**


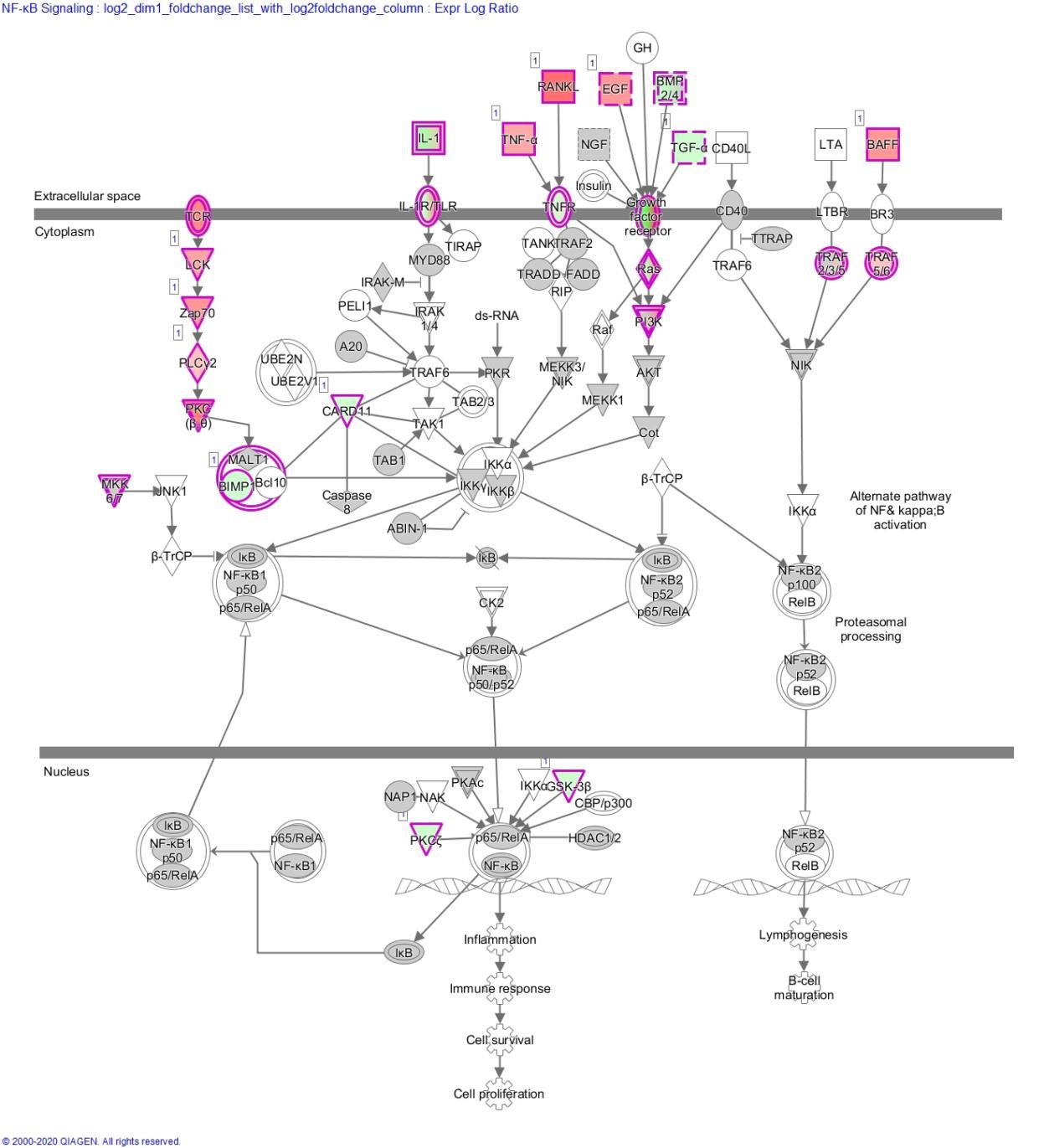


**Supplemental Figure 4. HIF1α Signaling Pathway**


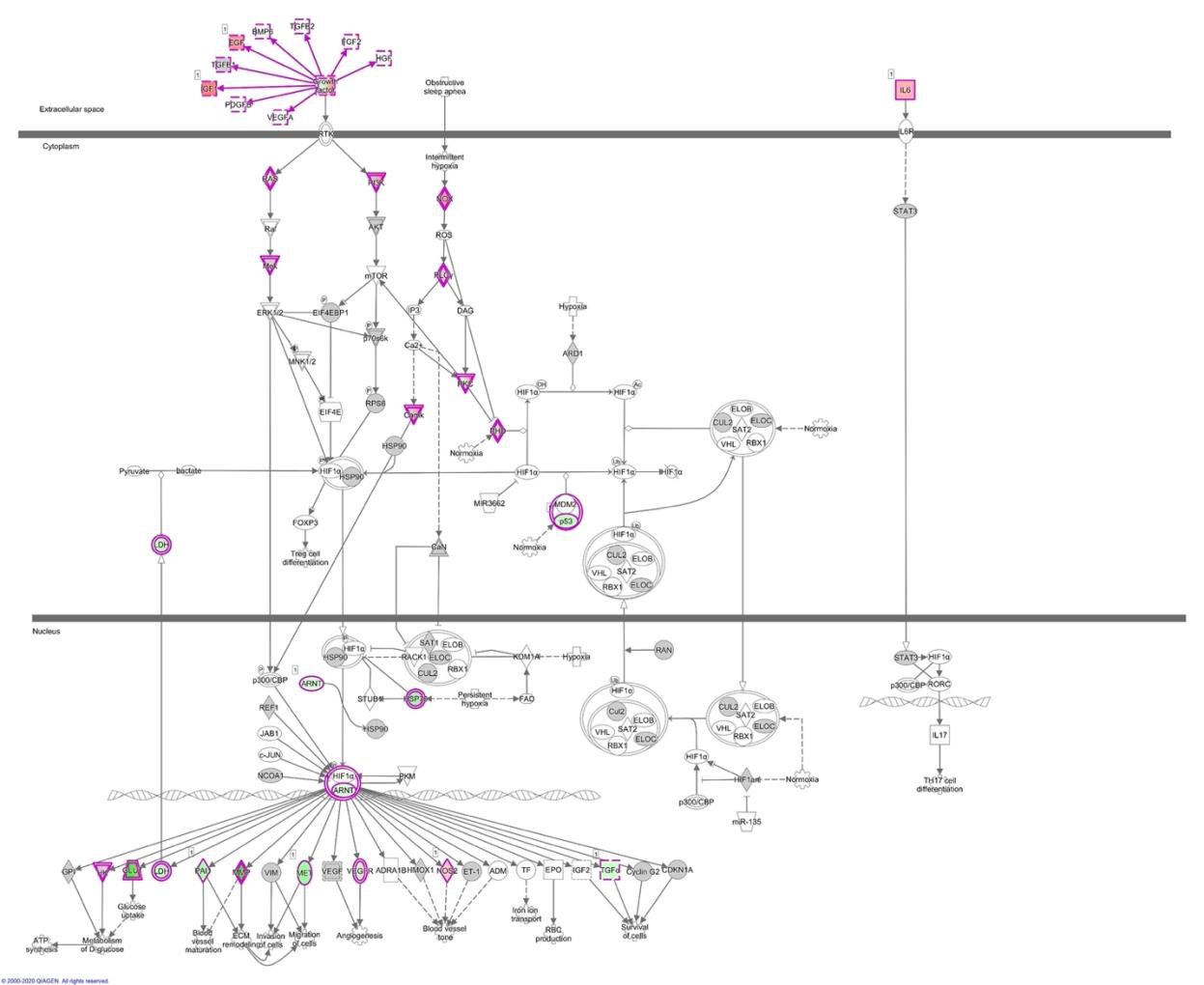


**Supplemental Figure 5. VEGF Family Ligand-Receptor Interactions pathway**


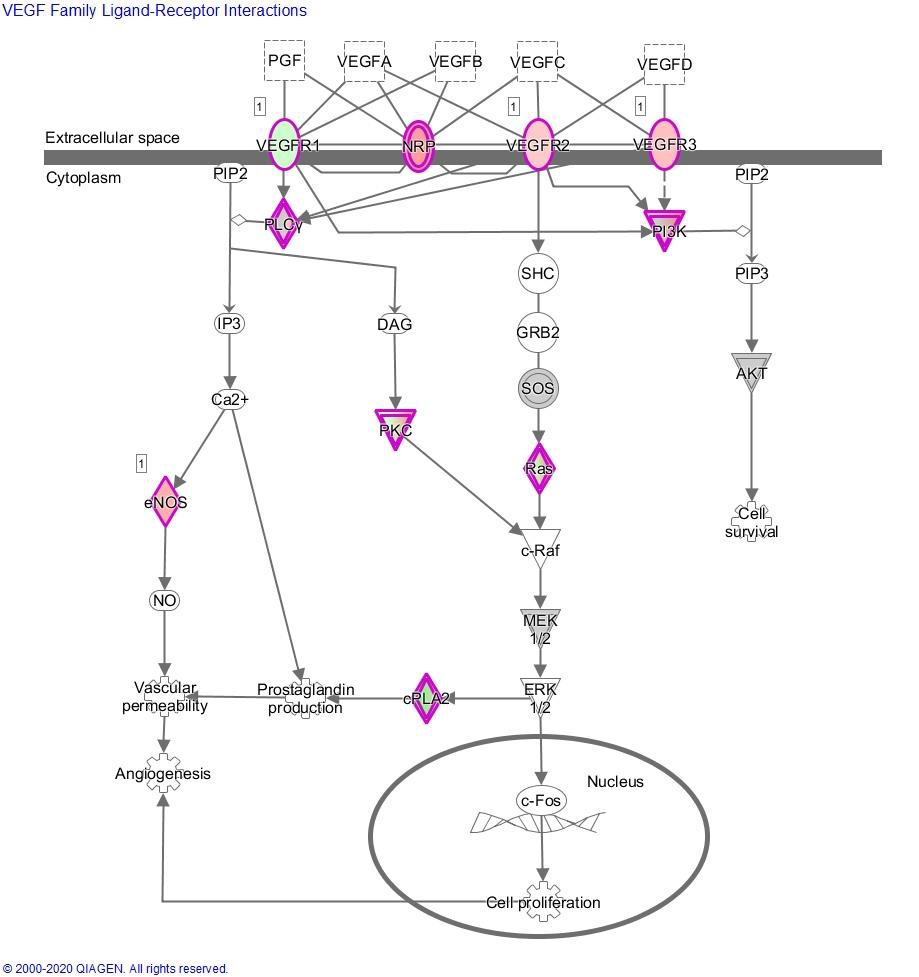


**Supplemental Table 3: Alignment Results via STAR**

|  | Number of input reads | Uniquely mapped reads % | % of reads mapped to multiple loci |
| --- | --- | --- | --- |
| Tumor 40 mouse reads | 14,930,832 | 89.64% | 8.65% |
| Tumor 42 mouse reads | 11,251,508 | 89.26% | 9.10% |
| Tumor 44 mouse reads | 17,761,793 | 91.48% | 6.87% |
| Average of Tumor mouse reads | 14,648,044 | 90.13% | 8.21% |
| Tumor 40 pig reads | 12,148,304 | 87.35% | 10.88% |
| Tumor 42 pig reads | 15,423,478 | 84.09% | 14.05% |
| Tumor 44 pig reads | 8,282,040 | 86.80% | 10.89% |
| Average of Tumor pig reads | 11,951,274 | 86.08% | 11.94% |

**Supplemental Table 4: Top 5 Diseases and Disorders enriched for genes significantly contributing to principal component 1**

| Name | Benjamini-Hochberg adjusted p-value range | number of genes |
| --- | --- | --- |
| 1. Cancer | 5.19E-112 – 4.68E-88 | 2948 |
| 2. Organismal Injury and Abnormalities | 5.19E-112 – 4.68E-88 | 3012 |
| 3. Dermatological Diseases and Conditions | 5.03E-98 – 4.68E-88 | 1930 |
| 4. Gastrointestinal Disease | 8.53E-77 – 3.47E-28 | 2700 |
| 5. Endocrine System Disorders | 4.6E-73 – 3.47E-28 | 2585 |

**Supplemental Table 5: Top 5 Molecular and Cellular Functions enriched for genes significantly contributing to principal component 1**

| Name | Benjamini-Hochberg adjusted p-value range | number of genes |
| --- | --- | --- |
| 1. Cellular Movement | 4.15E-56 – 3.47E-28 | 911 |
| 2. Cell-To-Cell Signaling Interaction | 7.67E-48 – 3.47E-28 | 774 |
| 3. Lipid Metabolism | 8.31E-34 – 3.04E-22 | 499 |
| 4. Molecular Transport | 8.31E-34 – 3.04E-22 | 870 |
| 5. Small Molecule Biochemistry | 8.31E-34 – 3.04E-22 | 558 |

**Supplemental Table 6: Top 5 Physiological System Development and Functions enriched for genes significantly contributing to principal component 1**

| Name | Benjamini-Hochberg adjusted p-value range | number of genes |
| --- | --- | --- |
| 1. Immune Cell Trafficking | 5.71E-55 – 3.47E-28 | 569 |
| 2. Tissue Morphology | 3.92E-54 – 3.47E-28 | 929 |
| 3. Hematological System Development and Function | 6.49E-52 – 3.47E-28 | 822 |
| 4. Cardiovascular System Development and Function | 4.24E-50 – 3.47E-28 | 626 |
| 5. Organismal Development | 1.15E-47 – 3.47E-28 | 1255 |

**Supplemental Table 7: Top 5 Diseases and Disorders enriched for genes contributing to principal component 2**

| Name | Benjamini-Hochberg adjusted p-value range | number of genes |
| --- | --- | --- |
| Cancer | 1.18E-11 – 2.94 E-04 | 243 |
| Organismal Injury and Abnormalities | 1.18E-11 – 2.94 E-04 | 246 |
| Dermatological Diseases and Conditions | 1.22E-09 – 2.94 E-04 | 191 |
| Endocrine System Disorders | 2.12E-09 – 2.94 E-04 | 221 |
| Gastrointestinal Disease | 6.16E-09 – 2.94 E-04 | 226 |
